# Impact of Age-Related Vision Changes on Driving

**DOI:** 10.3390/ijerph17207416

**Published:** 2020-10-12

**Authors:** Sonia Ortiz-Peregrina, Carolina Ortiz, Miriam Casares-López, José J. Castro-Torres, Luis Jiménez del Barco, Rosario G. Anera

**Affiliations:** Laboratory of Vision Sciences and Applications, Department of Optics, University of Granada, 18071 Granada, Spain; soniaortiz@ugr.es (S.O.-P.); clmiriam@ugr.es (M.C.-L.); jjcastro@ugr.es (J.J.C.-T.); ljimenez@ugr.es (L.J.d.B.); rganera@ugr.es (R.G.A.)

**Keywords:** aging, older drivers, driving performance, visual function, straylight

## Abstract

Aging leads to impaired visual function, which can affect driving—a very visually demanding task—and has a direct impact on an individual’s quality of life if their license is withdrawn. This study examined the associations between age-related vision changes and simulated driving performance. To this end, we attempted to determine the most significant visual parameters in terms of evaluating elderly drivers’ eyesight. Twenty-one younger drivers (aged 25–40) were compared to 21 older drivers (aged 56–71). Study participants were assessed for visual acuity, contrast sensitivity, halos, and intraocular straylight, which causes veiling luminance on the retina and degrades vision. Driving performance was evaluated using a driving simulator. The relationships between simulated driving performance and the visual parameters tested were examined with correlation analyses and linear regression models. Older drivers presented impairment in most visual parameters (*p* < 0.05), with straylight being the most significantly affected (we also measured the associated effect size). Older drivers performed significantly worse (*p* < 0.05) in the simulator test, with a markedly lower performance in lane stability. The results of the multiple linear regression model evidenced that intraocular straylight is the best visual parameter for predicting simulated driving performance (R^2^ = 0.513). Older drivers have shown significantly poorer results in several aspects of visual function, as well as difficulties in driving simulator performance. Our results suggest that the non-standardized straylight evaluation could be significant in driver assessments, especially at the onset of age-related vision changes.

## 1. Introduction

The overall aging of the population is leading to an increase in the number of older drivers. In the coming decade, around 95% of people aged 65 or over will be active drivers [1]. Although the data suggest that young drivers are involved in more traffic accidents per licensed driver [2], divers aged over 65, approximately, are known to experience an increase in accidents per km driven [3,4]. Moreover, older drivers are more likely to be considered at-fault [2]. This age group is over-represented in accidents at intersections or those caused by a failure to yield the right of way or properly respond to traffic signs and hazards [2]. Consequently, the design of more comprehensive methods for assessing driving skills and identifying unsafe drivers among the elderly is becoming increasingly important.

Vision is the fundamental sensory mechanism used when driving [5,6]. Aging triggers a series of changes in the ocular structures, including a marked loss of lens transparency, which causes a significant increase in light scattering by the eye’s optical media [7]. Intraocular scattering produces a veil of straylight over the retina; this is known to cause disability glare due to a loss of retinal image contrast [8,9,10]. Severe impairments in contrast sensitivity are known to be significantly associated with at-fault accident involvement [9]. Furthermore, closed-road studies have shown that contrast sensitivity is a significant predictor of driving performance [11,12]. Other findings demonstrate the fact that glare worsens a driver’s ability to adopt adequate safety margins [12]. Although visual acuity is the standard test for licensing purposes, it only presents a weak correlation with safety risk in older drivers [13]. In terms of driving performance, visual acuity does not give an accurate prediction of a driver’s ability to recognize hazards in all hazy conditions [14] or light levels [11].

In healthy eyes, straylight remains relatively constant up to the age of 45 but increases substantially with aging; it doubles by the age of 65 and triples by the time a person reaches 77 [10]. Besides greater glare, this increase can have various effects on vision quality such as the development of recognition difficulty and spatial orientation problems, as well as a loss of contrast or color vision [9,15]. However, despite these negative impacts, increased straylight may have a limited influence on visual acuity, as it does not deteriorate in the same way due to aging or eye diseases such as cataracts [15]. These two measures of visual function are therefore independent and should be assessed and considered separately; otherwise, if an older driver suffers from increased straylight, it may be misinterpreted if visual acuity (VA) is not impaired. As mentioned above, straylight is one of the main causes of glare complaints and clearly compromises contrast sensitivity in older drivers, even in photopic conditions [16]. Indeed, various studies have argued that straylight measurements should be included in driver eyesight tests [17,18,19] and proposed a straylight limit cutoff value for driving [20]. However, these proposals are assumptions based on self-reported hazard perception data [19,20] rather than objective information compiled from driving performance or accident data. Therefore, at the time of writing, there is still a lack of knowledge regarding the relationship between straylight and driving capacity or safety risk.

The present study aimed to examine the associations between the different parameters that characterize visual function and simulated driving performance, and determine which parameters are the most significant when evaluating driver eyesight, especially in older drivers.

## 2. Materials and Methods 

### 2.1. Participants

The study enrolled 21 younger drivers aged 25–40 (mean 29.8 ± 4.4 years) and 21 older drivers aged > 55 (mean 62.3 ± 4.3 years; range 56–71). All participants were recruited from the general population via an online campaign on our website. All participants were in good general health, had normal visual acuity (better than 20/25 or 6/7.5), had no eye diseases (no cataracts were present) or IOLs (intraocular lenses), had never previously undergone eye surgery, and were licensed drivers who drove regularly. Any participants who showed symptoms of simulator sickness (vertigo, nausea, dizziness, or sweating) were excluded from the study. Sociodemographic and driving characteristics data were collected using a brief questionnaire. The study was approved by the University of Granada’s Human Research Committee. Before participating in the study, all subjects were informed of the objectives and procedures and signed informed consent forms in accordance with the tenets of the Declaration of Helsinki. 

### 2.2. Vision Assessment

Visual acuity (VA) was measured using the POLA VistaVision^®^ (DMD Med Tech srl., Torino, Italy) Visual Acuity Chart at 5.5 m [log Minimum angle of resolution (MAR) scale] and contrast sensitivity (CS) was measured with the CSV-1000 test (Vector Vision, OH, USA) under the recommended viewing conditions (log units). The CSV-1000 chart has four rows for four different spatial frequencies: 3, 6, 12, and 18 cycles/degree (cpd). Each row contains eight vertical pairs of circles, of which one circle is blank and the other contains the sinewave patch. Each spatial frequency is presented at eight different contrast levels that decrease from left to right [21]. We also evaluated the visual discrimination capacity, which can be used to quantify patient-perceived visual disturbances, using Halo v1.0 software (http://digibug.ugr.es/handle/10481/5478; Laboratory of Vision Sciences and Applications, University of Granada, Granada, Spain). This test involves showing subjects a central high-luminance stimulus over a dark background, then peripheral stimuli are progressively shown at different positions and distances around the main central stimulus. Subjects have to press a button on the mouse every time they perceive a peripheral stimulus. The test generates a visual disturbance index (VDI), which ranges from 0 to 1 and provides information on the halo perceived by subjects. The greater the index, the lower the visual discrimination capacity and, therefore, the greater the halo effect [8,22]. This test has been successfully applied in basic and clinical research [22,23,24]. All measurements were made binocularly. 

Intraocular straylight was also measured monocularly using a C-Quant device (Oculus DG, Germany). The C-Quant is a computerized straylight meter that employs a compensation comparison method involving a series of concentric rings. The smallest ring—the test field—is divided into two halves and subjects are asked to look at this, while a concentric ring—representing the straylight source—flickers with a varying intensity and frequency. The flickering of the straylight source induces a certain amount of perceived flickering in the test field. Subjects are asked to compare the two halves of the test field and press a button to indicate which side flickers more clearly/strongly [25]. The results were expressed as logarithm straylight (log(s)), whereby higher values indicate a greater straylight effect [18,26].

If applicable, participants wore the same optical correction as during normal driving. 

### 2.3. Driving Simulator

We used the fixed-based SIMAX DRIVING SIMULATOR v4.0.8 BETA (SimaxVirt, Pamplona, Spain) with a 180° field of view to perform objective driving performance assessments. Participants drove along a route approximately 12.5 km long. The route comprised three main sections with different degrees of difficulty (a dual carriageway, a mountain road, and an inner-city circuit). The first section was a 4.5-km-long dual carriageway (two lanes of traffic in each direction), with a speed limit of 120 km/h, no buildings, moderate traffic, and a gentle curve. The second section was a winding, 6-km-long, single-carriageway mountain road with a variable speed limit of 40–90 km/h, no buildings, and moderate traffic. The third section was a 2-km-long inner-city circuit with a speed limit of 40–50 km/h, several intersections or roundabouts with traffic signals, many buildings, 16 pedestrians, and moderate traffic. All participants drove in a scenario with identical traffic flow and the same number of peripheral events as above. More information on this simulated environment can be found elsewhere [27,28]. Participants received at least two training sessions separated by a seven-day washout period. Each training session took about 20 min to complete when adhering to the established speed limits. At the end of the training sessions, which involved similar scenarios to the one used in the experimental session, all subjects said they felt totally comfortable with the equipment. Participants were instructed to drive as they would normally do, respecting the rules of the road. They were not asked to give any specific responses during the driving task. 

We analyzed the following variables to assess driving performance: mean speed, standard deviation of the lateral position (SDLP, a measure of steering stability), distance travelled while encroaching the hard shoulder, distance travelled while encroaching the opposite lane, total distance driven outside the driver’s lane (i.e., the total distance travelled encroaching the opposite lane and hard shoulder), the standard deviation of the steering wheel angular velocity (a measure of how demanding the steering task was), and the total time required to complete the circuit. As a measurement of general driving performance, the overall driving performance score (ODPS) was obtained. This composite score was calculated by obtaining z-scores for each driving performance parameter before calculating a mean z-score for each participant, as per other works [29,30,31]. Z-scores are a measurement of how many standard deviations an individual value is away from the group mean. The scores were transformed so that positive z-scores represented a better performance and negative scores a worse performance than the mean. We considered the following individual variables when calculating the ODPS: distance travelled while encroaching the hard shoulder, SD of the steering wheel angular velocity, SDLP, total distance driven outside the driver’s lane, and total time. We did not include the mean speed because we considered that it is not indicative of driving performance quality, in line with other studies [28,30,31]. For the mountain road section, we ignored the distances travelled while encroaching the opposite lane and the hard shoulder because these distances are accounted for in the total distance driven outside the driver’s lane. However, we did incorporate the total time required as per Wood [30], who established that higher times indicated a poorer performance given that these drivers needed more time to detect all the stimuli presented along the road, which could mean they find it harder to complete the route correctly. Although visually impaired drivers adopt slower speeds, this is not sufficient to allow them to perform these driving-related tasks at the same level as people with no visual impairment [14,30]. Although some of the individual variables may be more important than others in terms of safety, we weighted them equally when calculating the ODPS as there is a lack of convincing evidence supporting the use of differential weighting [30].

### 2.4. Data Analysis

Statistical analyses were performed using SPSS 24.0 (SPSS Inc., Chicago, IL, USA) and statistical significance was set at *p*-values <0.05. All continuous variables were calculated as mean ± SD and categorical variables as percentages. Differences between the two groups for all vision and driving performance variables were examined with the independent t-test and the Mann–Whitney U test for variables with a non-normal distribution. Statistics and p-values are reported, as well as the effect sizes, which were obtained with GPower software. The relationship between visual function and driving performance was first explored with a bivariate correlation analysis, including the ODPS and all the visual parameters studied. To this end, we used Pearson and Spearman correlations for nonnormally distributed variables. Any visual parameters that correlated significantly with the ODPS were included as possible predictors or independent variables in a forward stepwise multiple linear regression model, with the ODPS as the dependent variable. We also checked regression model assumptions and found that none of them were violated. This procedure has been used successfully in a previous study that analyzed the influence of other visual parameters on drivers of different ages [30].

## 3. Results

Table 1 shows the demographic data for the entire sample and the two age groups. The sample included mainly men (~90%), with similar percentages in both age groups. All drivers had at least three years’ experience and most drove daily or several times a week. The younger group rated their own driving ability at a slightly higher level than did the older drivers, but no participants rated their driving as fair or poor. No participants reported having had a traffic accident in the past year.

### 3.1. Visual Function 

Table 2 shows the results (mean ± SD) of the visual assessment for the two age groups. Analysis of the group comparison revealed an age-related visual impairment in all the parameters studied. Visual acuity is the only parameter that did not differ significantly between groups, showing a reduction of just 0.03 log MAR units in older compared to younger drivers. The older group returned poorer results for the remaining parameters, with a 9.5% reduction in contrast sensitivity and an increase of more than 100% in VDI. In addition, straylight levels were significantly higher in the older group, as they presented a 33% increase over the younger drivers. This indicates a greater influence of scattered light in the eye media and, therefore, more glare sensitivity in older drivers.

### 3.2. Driving Performance

Table 3 gives the mean driving performance data for the two groups. On the dual carriageway, both groups drove at similar average speeds. With regard to steering stability, older drivers showed a 50% higher standard deviation (SD) in steering wheel angular velocity. This indicates that older group requires a greater steering effort and consequently they find it harder to keep the car in the correct lane position, which is supported by the fact that they drove on the shoulder for greater distances than the younger group (~248% difference). On the mountain road, older drivers again demonstrated more difficulty in maintaining correct lane position, with a 27% higher standard deviation of the lateral position (SDLP). Therefore, this group travelled significantly longer total distance outside their lane (~160%). Similarly, the SD of the steering wheel angular velocity was 28% higher for the older group in the mountain section. Driving through the inner-city circuit, the SD of the steering wheel angular velocity was approximately 17% higher for the older drivers. Also, older participants drove significantly slower in this section, a result that contrasts with those obtained for the rest of the circuit, where the two groups drove at similar speeds. The city is a more complex environment, with more visual stimuli (traffic signs, traffic lights, etc.), and more variation of road users (traffic, parked cars, and pedestrians). This could indicate that drivers perceive different parts of the route as having different degrees of difficulty and adopt compensatory mechanisms to mitigate the increase in perceived risk. 

Finally, older drivers required significantly more time to complete the full route (~8%). Furthermore, the ODPS indicated that younger subjects generally had better driving performance, achieving a significantly higher average score (Table 3).

### 3.3. Relationship between Age-Related Visual Impairment and Driving Performance

Bivariate correlation analysis showed that worse scores for the visual variables studied correlated significantly with a poorer driving performance. The closest correlation was with the straylight parameter (r = −0.652; *p* < 0.001), and the weakest was with binocular visual acuity (ρ = −0.322; *p* = 0.038). By contrast, the correlations between ODPS and contrast sensitivity (r = 0.447; *p* = 0.003) and the visual disturbance index (ρ = −0.471; *p* = 0.002) were very similar. 

**Figure 1:**
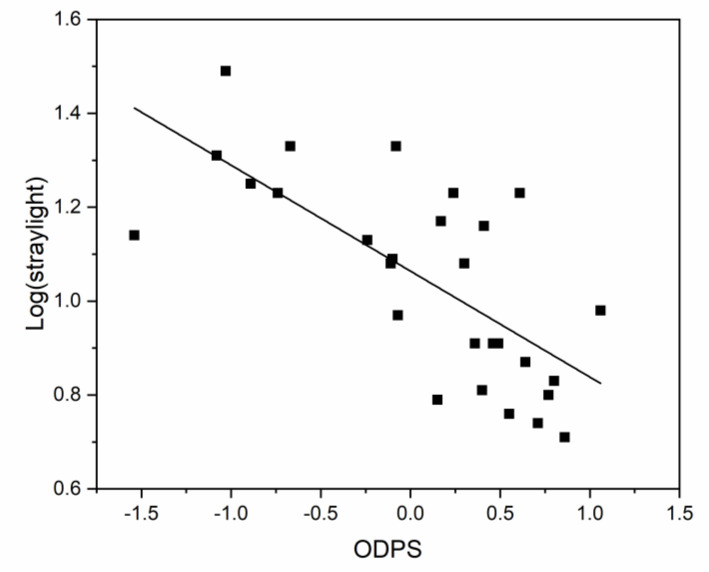
Scatterplot of overall driving performance score (ODPS) and the visual parameter selected as a predictor using the regression model (log(s)).

Finally, the multiple linear regression model, taking the ODPS as the dependent variable and the visual parameters as possible predictors, only returned the straylight parameter as a significant predictor of driving performance (ODPS). Figure 1 is a scatterplot of the ODPS against log(s) that reveals the relationship between the two parameters. The model indicated that the variable log(s) can predict 51.3% of the variance in the ODPS (R^2^ = 0.513; multiple r = 0.716). The equation for the model (Equation (1)) is shown below (standard errors are given in brackets):

ODPS = 2.547 − 2.508 log(s)_(0.447)_ (1)

## 4. Discussion

Older drivers were found to have diminished visual acuity, poorer contrast sensitivity, and were also influenced more by glare and halo. This group also showed a significantly higher straylight level. This general impairment of visual function has already been reported in previous studies on aging [8,10,32]. As a consequence of these changes, older populations experience a decline in the skills needed to perform certain tasks, such as driving, thus increasing the risk of accidents. This often causes older drivers to self-regulate their driving, or even stop driving all together. This decision is known to have a negative impact on health, as it leads to a higher prevalence of depression, social isolation, and poorer access to health services, which diminishes older people’s quality of life [33].

In this context, our results demonstrate that older drivers drive less efficiently, presenting a higher SDLP and driving longer distances outside their lane, even though they try to correct the vehicle’s position, as evidenced by the higher SD of the steering wheel angular velocity. These results are in general agreement with previous studies, which found that older drivers have a greater incidence of lane excursions [34], more difficulties changing lane [35], and less control over the vehicle’s position in unexpected events or divided attention tasks [27]. The reduced visual capacity observed in older drivers could be responsible for most of these changes, as it affects visual guidance abilities and visual attention span, making older drivers slower and less accurate when detecting all the stimuli on the road [36]. Consequently, older drivers have a poorer overall driving performance [30]. However, they may be aware of this decline, as our results show that this group significantly reduced their speed in the city, the environment with the highest visual demand, perhaps as a compensatory mechanism. Complex road environments, like urban scenarios, are known to influence speed adaptation behavior [37]. Older drivers usually reduce their speed to mitigate the perceived risk due to either the environment or their visual, cognitive, or motor decline [35]. Nevertheless, according to our results, it has been suggested that reducing driving speed is not enough to eliminate any errors in road sign recognition and hazard avoidance [14], nor does it improve performance in terms of driving measures (e.g., driving outside the lane) in older drivers with eye disease [28] or when distracted [27]. 

The correlation analysis showed that a decline in any of the visual parameters we analyzed correlated with a worse overall driving performance (lower ODPS). Visual acuity has been reported to correlate with real driving recognition tasks [14]. However, the correlation with crash data is much weaker [13] or even insignificant, which could indicate that poorer visual acuity does not necessarily imply a higher risk of accidents [38]. Conversely, it is known that reduced contrast sensitivity is critical for safety risk, as drivers involved in crashes are up to eight times more likely to have severe contrast sensitivity impairment [9]. With regard to driving performance, our study found a significant correlation with CS, similar to that observed in a previous closed-road study [30]. The complex driving environment includes low-contrast stimuli, such as deteriorated road markings, signals, and bumps, which may be harder to detect for visually impaired older drivers. The ability to detect these stimuli could be critical and a visual acuity test may not provide an adequate assessment of the appropriate visual function parameters [14,20].

Other studies have found that some nonstandard visual tests can predict driving performance. In our study, the group of older drivers, who suffer a greater influence from halos and glare, obtained significantly lower ODPS. Kimlin et al. [29] demonstrated that a wider halo area in older drivers was associated with a poorer overall nighttime driving performance. Additionally, Gray and Regan [12] found that the presence of glare due to low-sun conditions resulted in riskier behavior during left turns, affecting older drivers to a greater extent. With respect to sign legibility, Dewar et al. [39] noted that only older drivers presented a reduced legibility distance in the presence of glare. In one of our recent studies concerning older drivers with and without cataracts, we found significant correlations between greater halo perception and increased difficulty maintaining the car within the lane and a worse general driving performance [28]. Similarly, in this study, we found that drivers with poorer visual discrimination perform worse in the driving simulator, particularly in terms of lane stability. This may be explained by the fact that poorer visual discrimination and glare sensitivity due to straylight effects could hamper driver discrimination and recognition of road cues and other stimuli, thereby causing difficulties while driving.

Straylight is problematic in daytime environments, causing difficulties with skills such as recognition or spatial orientation, something subjects often refer to as “hazy vision.” This is of particular interest given that these complaints are also relevant when driving, a task in which recognizing road signs and markings, correct spatial location within a lane, and respect for other road elements and user are all paramount. One of our recent studies on the effect of driver distraction in different age groups showed a significant correlation between straylight and simulator driving performance. In said study, the group of older drivers with higher straylight values found it harder to maintain the vehicle within the lane and experienced more collisions in the sessions both with and without distractions [27]. In another recent study, straylight was reported to be greater when under the influence of alcohol and presented a significant association with driving performance in normal conditions and under the influence of alcohol [40]. Moreover, it is worth noting that the effects of straylight during nighttime driving could complicate driving for older drivers with no eye disease, even in low-beam glare conditions [20]. Our results are consistent with those of previous studies [17,20,28] and suggest that intraocular straylight could be a particularly useful visual parameter to measure when assessing the fitness to drive of older drivers, although this conjecture requires further research. 

In support of the aforementioned idea, our regression model selected straylight as the only visual parameter for predicting driving performance, accounting for 51.3% of the variance in the ODPS. This visual test attains a prediction percentage that is slightly higher than the value reported in an on-road study that combined other visual parameters [30], and is also much higher than another work with measurements relating to crash data [13]. Our results are also in line with the conclusions of Bal et al. [18], whose study highlighted the importance of measuring straylight and its applicability for inclusion in visual skill assessments for licensing purposes. Another study, which evaluated the vision of 2422 European drivers, showed that a significant percentage of older drivers had straylight levels that could imply an impaired driving performance (29.5%). However, the percentage that did not achieve the minimum visual function level included in the current standards, such as visual acuity and visual field, was much lower. (5.3% and 2.4%, respectively) [19]. Although these studies defined straylight levels with the potential to impair driving performance as having a log(s) > 1.4, our results show significantly decreased driving performance at lower values. In addition, it is worth noting that the older drivers in our study had very good visual acuity, double the legal minimum required for driving. The varying prevalence of impaired VA and straylight levels shows that these two visual functions are mutually independent. An earlier study demonstrated that the two parameters change independently in a normally aging and cataractous eye [15]. Both parameters explored independent aspects of visual quality and lent support to the suggestion that VA is not the only significant parameter in a fitness-to-drive assessment, particularly in the case of older drivers.

Our results indicate that we require more in-depth knowledge of the role of ocular straylight in driving and a review of what represents a safe level when behind the wheel. For instance, comparisons between older drivers with different levels of intraocular scattering would help support our results. However, we could not make such a comparison because of the limited sample size and age range of our older drivers’ group, which supposes an important limitation that will be taken into account in future works. Nevertheless, our previous study in two age-matched groups of older drivers (with and without eye diseases) also demonstrated that scattering-related visual measures can be significant predictors of driving ability [28]. On the other hand, we also need to determine the relationship between intraocular straylight and actual driving based on on-road performance, since our results were obtained for simulated driving in daytime conditions and must therefore be interpreted with caution. Furthermore, due to the frequency with which older drivers report simulator sickness [41], in future studies we believe it would be interesting to try to adapt the environment and design of the driving simulator task in order to limit this occurrence (e.g., reduce the number of turns and roundabouts along the route). However, the relative validity of driving simulators with respect to real-world driving has been demonstrated elsewhere [42], indicating that their use represents a well standardized method and the most ethical way of conducting studies as they ensure a safe environment for the driver in all situations. It would also be interesting to study straylight levels during nighttime driving, where glare sensitivity could have an even greater effect.

## 5. Conclusions

To the best of our knowledge, this study is the first to analyze the importance of intraocular straylight as a predictor of driving performance, looking at driving-related tasks in an objective manner. Our study helps support previous reports of a possible relationship between straylight and driving performance. These results demonstrate that straylight could be a valuable indicator of a driver’s general visual status, especially when aging begins to affect vision. Although the older drivers in our sample had good eye health and visual acuity, they had experienced significant vision changes, and these correlated with poorer driving abilities. More investigation is needed to confirm these results and analyze the impact of different levels of straylight on actual driving performance in different environments and conditions and to study the potential benefit of including a straylight assessment, especially when testing fitness to drive in older drivers. 

## Figures and Tables

**Table 1:** Demographic characteristics for the total sample and the two age groups.

Item	Younger (Aged 25–40)	Older (Aged >55)	Total Sample
Age (years) (mean ± SD)	29.8 ± 4.4	62.3 ± 4.3	46.1 ± 17.0
Gender			
Male	85.7%	95.2%	90.5%
Female	14.3%	4.8%	9.5%
Driving experience (years)			
between 0 and 3 years	0%	0%	0%
between 3 and 5 years	9.5%	0%	4.8%
>5 years	90.5%	100%	95.2%
Distance driven in the past year (km)			
<500	0%	0%	0%
500–999	14.3%	0%	7.7%
1000–4999	33.3%	16.7%	25.6%
>5000	47.6%	83.3%	64.1%
No answer	4.8%	0%	2.6%
Driving frequency			
Daily	42.9%	38.9%	41%
Several times/week	33.3%	55.6%	43.6%
Once a week	9.5%	5.5%	7.7%
2–3 times/month	14.3%	0%	7.7%
Once a month or less	0%	0%	0%
Self-perceived driving ability			
Excellent	19.0%	11.1%	15.4%
Good	61.9%	55.6%	59.0%
Normal	19.1%	33.3%	25.6%
Fair/Poor	0%	0%	0%

**Table 2:** Comparison of visual function measurements for the two age groups.

Visual Parameter	Younger (Aged 25–40)	Older (Aged > 55)	t/Z	*p*-Value	Effect Size (Cohen’s *d*)
Binocular VA (log MAR) *	−0.06 ± 0.06	−0.03 ± 0.06	−1.688	0.091	0.5
Binocular CS (log CS)	1.89 ± 0.10	1.71 ± 0.14	4.987	<0.001	1.48
Binocular VDI *	0.14 ± 0.03	0.32 ± 0.28	−3.292	0.001	0.90
Log(straylight)	0.89 ± 0.13	1.19 ± 0.11	−7.703	<0.001	2.49

VA, visual acuity; CS, contrast sensitivity; VDI, visual disturbance index; * Mann–Whitney U test.

**Table 3:** Group mean (± SD) driving performance outcomes for the two age groups.

Driving Performance Parameter	Younger (Aged 25–40)	Older (Aged > 55)	t/Z	*p*-Value	Effect Size (Cohen’s *d*)
Dual Carriageway					
Mean speed (km/h)	118.44 ± 7.81	114.64 ± 10.53	1.316	0.196	1.36
Distance driven while encroaching the hard shoulder (m)	72.98 ± 59.06	253.75 ± 215.45	−3.708	0.001	1.14
SD steering wheel angular velocity (rad/s)	0.16 ± 0.04	0.24 ± 0.06	−4.907	<0.001	1.57
Mountain Road					
Mean speed (km/h)	56.06 ± 2.25	56.59 ± 2.53	−0.698	0.490	0.22
SDLP (m)	0.51 ± 0.06	0.65 ± 0.13	−4.567	<0.001	1.38
Distance driven while encroaching the opposite lane (m)	212.20 ± 116.61	593.04 ± 378.13	−4.379	<0.001	1.36
Distance driven while encroaching the hard shoulder (m) *	20.35 ± 15.16	81.21 ± 74.29	−3.709	<0.001	1.14
Total distance driven outside the lane (m)	265.77 ± 139.78	693.23 ± 377.33	−4.853	<0.001	1.50
SD steering wheel angular velocity (rad/s)	0.63 ± 0.14	0.81 ± 0.26	−2.812	0.008	0.86
City					
Mean speed (km/h)	32.02 ± 4.88	29.96 ± 6.37	2.842	0.007	0.36
SD steering wheel angular velocity (rad/s)	1.13 ± 0.17	1.32 ± 0.17	−3.529	0.001	1.12
Total Circuit					
Total time (s)	884.51 ± 44.62	958.05 ± 99.38	−3.030	0.005	0.95
ODPS	0.53 ± 0.28	−0.50 ± 0.55	7.658	<0.001	2.36

SDLP, standard deviation of the lateral position; ODPS, overall driving performance score. * Mann–Whitney U test.
